# Atypical Complete Femoral Fractures Associated with Bisphosphonate Use or Not Associated with Bisphosphonate Use: Is There a Difference?

**DOI:** 10.1155/2016/4753170

**Published:** 2016-11-20

**Authors:** Sang-Min Kim, Youn-Soo Park, Young-Wan Moon, Seung-Hoon Kang, Ingwon Yeo, Seung-Min Oh, Seung-Jae Lim

**Affiliations:** ^1^Department of Orthopaedic Surgery, Seoul Medical Center, Seoul, Republic of Korea; ^2^Department of Orthopaedic Surgery, Samsung Medical Center, Sungkyunkwan University School of Medicine, Seoul, Republic of Korea; ^3^Department of Orthopaedic Surgery, Samsung Changwon Hospital, Sungkyunkwan University School of Medicine, Changwon, Republic of Korea

## Abstract

The purpose of this study is to compare clinical characteristics and surgical outcome of atypical complete femoral fractures associated with bisphosphonates (BPs) use and those of fractures not associated with BPs use. Seventy-six consecutive patients (81 fractures) who had been operatively treated for a complete atypical femoral fracture were recruited. Of the 81 fractures, 73 occurred after BPs medication of at least 3 years (BP group) while 8 occurred without a history of BP medication (non-BP group). There were no differences in demographic data and fracture- and surgery-associated factors between the two groups. Of 76 patients (81 fractures), 54 (66.7%) fractures showed bony union within 6 months after the index surgery and 23 (28.4%) showed delayed union at a mean of 11.2 months (range, 8–18 months). The remaining 4 fractures were not healed, even 18 months after the index surgery. There was no difference in healing rate between the BP group and the non-BP group. There were strong correlations between the fracture height and the degree of bowing regardless of BPs medication. All fractures except 1 occurred at the diaphyseal region of the femur when not associated with BP medication.

## 1. Introduction

Bisphosphonates (BPs) are known to inhibit osteoclast-mediated bone resorption, which may cause accumulation of trabecular microdamage [1] and compromise the mechanical and regenerative properties of bone, resulting in fractures [2, 3]. Long-term use of bisphosphonates (BPs) has been considered one of the causes of low-energy femoral fracture, and these have been termed atypical femoral fractures [3–9]. However, the association between long-term use of BPs and atypical femoral fractures is still unclear and lots of studies have showed variability of relative risk values [10–13].

In 2010, the American Society for Bone and Mineral Research (ASBMR) defined atypical femoral fractures with 5 major features including being located on subtrochanteric and diaphyseal region, medial spike or lateral cortex hypertrophy, transverse or short oblique configuration, and being not comminuted and after no or minimal trauma [14]. It was also noted that these fractures may occur even without use of BPs. ASBMR Task Force 2013 revised case definition of atypical femoral fractures, deleting the clause associated with the use of pharmaceutical agents such as BPs, glucocorticoids, and proton pump inhibitors [15]. Under this definition, stress fractures which have been well recognized to be caused by bowing deformity may also be classified as atypical femoral fractures [16].

Several recent studies have addressed the operative treatment, complications, and healing rates of atypical femoral fractures after BPs medication [7, 17–21]. Delayed or failed fracture healing is becoming a major concern after fracture stabilization in patients taking BPs [19]. However, there are limited data regarding atypical fractures occurring without use of BPs. Only a few cases have been reported to date. Therefore, this study was investigated to compare clinical characteristics and surgical outcome of atypical complete femoral fractures associated with BPs use and those of fractures not associated with BPs use.

## 2. Materials and Methods

From July 2008 to February 2014, a consecutive series of 86 patients (91 fractures) who had been operatively treated for a complete atypical femoral fracture were identified. Atypical femoral fractures were defined by characteristic radiographic findings according to the criteria of the 2013 ASMBR Task Force [15]. All fractures were located along the femur from just distal to the less trochanter to just proximal to the supracondylar flare and occurred with a history of minimal or no trauma. Minimal trauma was defined as a slip or fall from a standing height or less [22]. Of these 86 patients, 3 patients (3 fractures) who had a previous experience of fracture, infection, or surgery of the affected femur were excluded from this study, as were 2 patients (2 fractures) who had a history of corticosteroid use, hormonal treatment, rheumatoid arthritis, metabolic bone disease other than osteoporosis such as hyperparathyroidism, or paraneoplastic syndrome. No pathologic fractures-associated metastatic malignancies were found. Five patients (5 fractures) were lost to follow-up before bony union. The final cohort comprised 76 patients (81 fractures). Of the 81 fractures, 73 occurred after BPs medication of at least 3 years (BP group) while 8 occurred without a history of BP medication (non-BP group). Mean follow-up period was 32.4 ± 9.4 months (range, 12–62 months). This study was approved by our Institutional Review Board and informed consent was waived for this retrospective study. Prodromal symptoms developed in 21 (25.9%) of the 81 fractures and the mean duration of symptoms was 5.3 ± 2.3 months (range, 1–8 months). All patients discontinued BP medications at the time of admission to our hospital after drug verification.

For each patient, we retrospectively searched detailed demographic data including age, affected side, sex, body mass index (BMI), and bone mineral density (BMD). Comorbidities and functional status [23] of the study population are also investigated. From our fracture database, characteristics of fracture and surgery that include injury mechanism, fracture location, fracture height [8], bilaterality, operation time, and the time interval from injury to operation were identified. Fracture location was categorized into two groups (subtrochanteric and diaphyseal). All of the fractures were fixed with an intramedullary nail.

Standing femoral AP radiographs with correct patellar forwarding with no rotation and lateral radiographs with the medial and lateral condyle of the femur overlapping were selected for data analyses. Preoperatively, femoral neck-shaft angle [24] and coronal and sagittal bowing of the femur were evaluated. Femoral bowing was measured using the curvature of the opposite femur as previously described [22]. In cases of bilateral fractures, bilateral plain radiographs before the second fracture were available. With respect to the reduction status of fractures, coronal alignment of the femur was defined as valgus, neutral, or varus and sagittal alignment was defined as flexion, neutral, or extension. Fracture healing was defined as bony bridging of 3 or 4 cortices on anteroposterior (AP) and lateral radiographs [25]. Fractures which showed bony union more than 6 months after the index surgery were considered to achieve delayed union. Nonunion is defined as persistence of the fracture 12 months after initial trauma without any tendency to progressive union in the previous 3 months [26].

### 2.1. Statistical Analyses

Basic descriptive statistical analyses were used to describe the study population. Values were subjected to averaging or percentages with use of Statistical Package for the Social Science software version 18.0 (SPSS, Chicago, IL, USA). The Mann-Whitney test was used for continuous variables, and Fisher exact test was used for categorical variables. Statistical significance was defined as *p* ≤ 0.05. Spearman's correlation coefficient was used to examine the correlation between the fracture height and anterior or lateral femoral bowing.

## 3. Results

**Figure 1:**
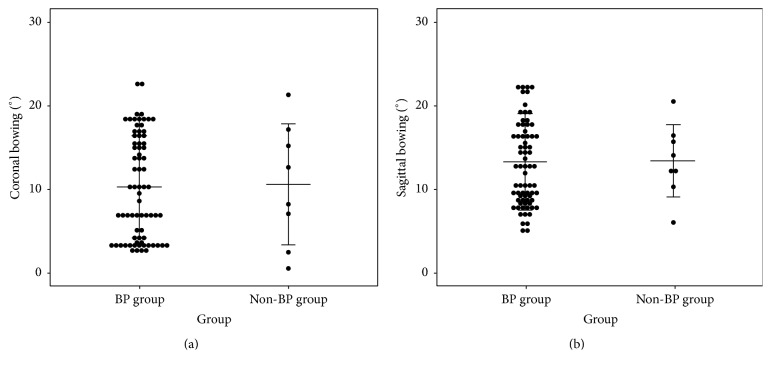
Comparison of (a) coronal and (b) sagittal bowing in patients of the BP group and the non-BP group. The mean, standard deviation, and data plots are shown.

Demographic data of the BP group and the non-BP group are summarized in Table 1. There were no significant differences between the two groups. Fracture- and surgery-associated factors of the BP and the non-BP groups are compared in Table 2. In the BP group, atypical fracture occurred at the subtrochanteric region in 27/73 (36.9%) while, in the non-BP group, only 1 of 8 (12.5%) occurred at this region. All fractures except 1 occurred at diaphyseal region when not associated with BP medication. However, statistical significance was not seen between the two groups. Radiographic results before and after surgery are presented in Table 3. Preoperative femoral bowing and reduction configuration during surgery showed similar patterns between the two groups (Figure 1).

**Figure 2:**
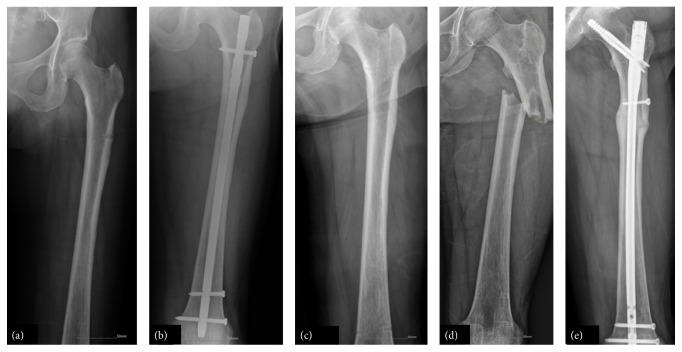
(a) Initial radiograph of a 64-year-old woman shows an atypical fracture of the subtrochanteric region of the femur. She had no history of antiosteoporotic medication. (b) Intramedullary nailing was performed. (c) After complete healing, implanted nail was removed 12 months after initial surgery. (d) Refracture occurred 33 months after nail removal. (e) A radiograph at 12 months after surgery shows complete healing.

**Figure 3:**
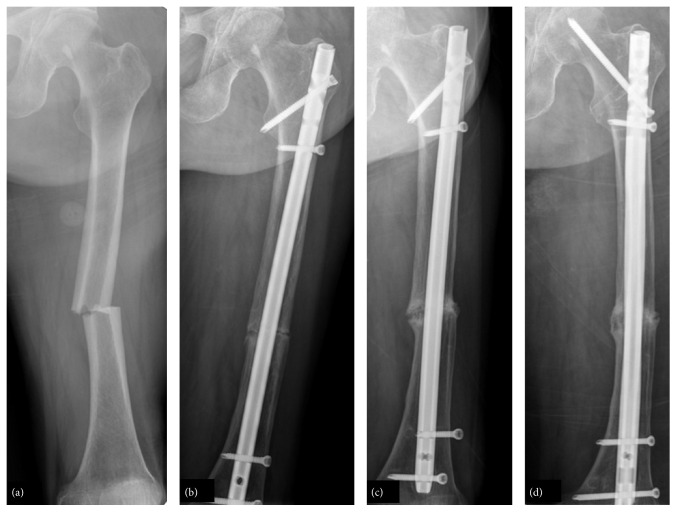
(a) A 77-year-old woman with a history of BP medication for 8 years experienced a complete femoral fracture after slip-down. (b) At 6 months after surgery, the fracture site was not healed. (c) She went on to develop nonunion with loosening of a distal interlocking screw even 3 years after surgery. (d) Revision surgery with a thicker and longer nail was performed.

Of 76 patients (81 fractures) in the final cohort, 54 (66.7%) fractures showed bony union within 6 months after the index surgery. Of the BP group (73 fractures) 49 achieved complete union within 6 months after surgery while of the non-BP group (8 fractures) 5 achieved complete union within 6 months after surgery (*p* = 1.000). Of 81 fractures, 23 (28.4%) yielded delayed union at a mean of 11.2 months (range, 8–18 months), but there was no difference between the two groups (20/73 versus 3/8, *p* = 0.682). The remaining 4 fractures were not healed, even 18 months after the index surgery. Of these 4 fractures, 2 received revision osteosynthesis (Figure 2) and 1 received total hip replacement; 1 patient refused reoperation up to the final follow-up. Among 8 of the non-BP group, 1 experienced refracture and underwent revision surgery. After she obtained complete union, implanted nail was removed. However, refracture occurred at the same region (Figure 3).

**Figure 4:**
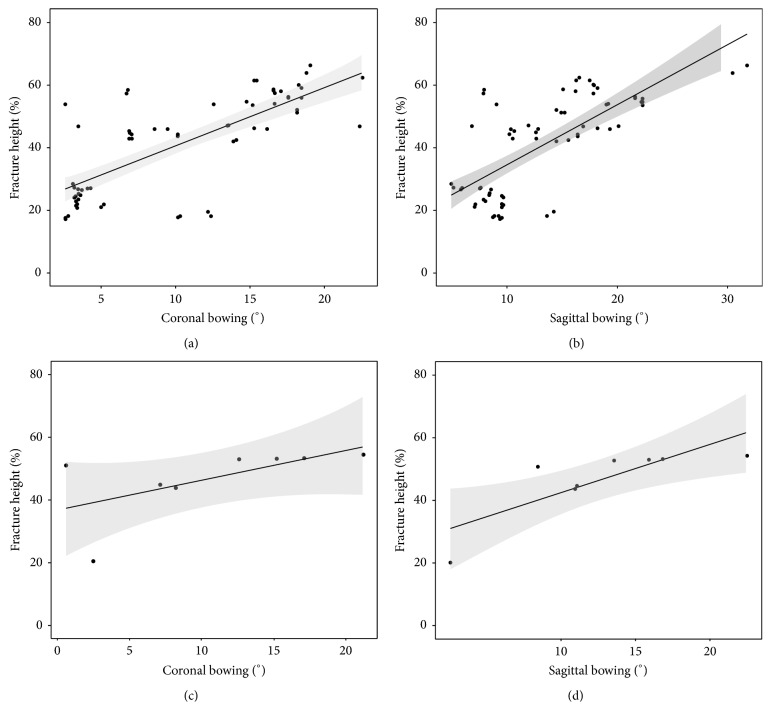
Scattered diagram showing the relation between the fracture height and the femoral bowing (a) in the coronal plane (Pearson's coefficient of 0.728) and (b) in the sagittal plane (0.665) of the BP group and (c) in the coronal plane (0.833) and (d) in the sagittal plane (0.929) of the non-BP group. Linear line; approximate line, gray zone; 95% prediction intervals.

Correlation analysis results between the fracture height and the coronal/sagittal bowing are shown in Figure 4. There were positive correlations between the fracture height and the coronal/sagittal bowing in both the BP group (*r* = 0.728 and *r* = 0.665, resp.) and the non-BP group (*r* = 0.833 and *r* = 0.929, resp.).

## 4. Discussion

After the concept of atypical femoral fractures became known widely, in Asian countries especially, stress fractures associated with bowing deformity have been confused with atypical fractures associated with long-term use of BPs [16]. According to ASMBR 2013, stress fractures, which occur without a history of BPs medication may also be classified as atypical femoral fractures. In our series, 68 patients (73 fractures) with a history of BPs medication of at least 3 years (BP group) and 8 patients (8 fractures) without a history of BP medication (non-BP group) are recruited. Tan et al. [27] reported the rate of atypical femoral fractures without BP use was 8% in their series of 50 patients who fulfilled distinctive radiographic characteristics of the atypical fracture.

Recently, there is fast growth in knowledge regarding atypical fractures associated with long-term BP use and our improved understanding of them could be beneficial to prevent functional decline and deconditioning in the elderly. However, we have little information about atypical fractures occurring without a history of BPs medication. It is still unclear whether clinical characteristics and surgical outcome of atypical complete femoral fractures without a history of BP use are similar to those of fractures associated with long-term use of BPs. In the present study, there were no differences in demographic data and fracture- and surgery-associated factors between the two groups.

Of 76 patients (81 fractures) in the final cohort, 54 (66.7%) fractures showed bony union within 6 months after the index surgery and 23 (28.4%) showed delayed union at a mean of 11.2 months (range, 8–18 months). A previous study [18], which evaluated 41 atypical, low-energy femoral fractures associated with ≥5 years of BP, reported that 98% (40/41) showed radiographic union at a mean of 8.3 months (range, 2–18 months). Healing times of almost 8 months for these fractures seemed to be longer than those for typical femoral fractures, which heal at an average of 3 to 6 months. Our findings also showed that there was no difference in healing time between the BP group and the non-BP group.

Asians have differences in femur geometry with higher rates of bowing, compared to western populations [5]. Some authors suggested that stress concentration on the diaphysis due to anteriorly and laterally bent shape of the femur may be an important causative factor of the atypical fracture [8, 16, 22]. Mechanical alterations due to the bowing of the femur increase the bending force on the anterior and lateral cortex. Moreover, the progression of this deformity shifts the area of maximal tensile stress to a more distal site of the diaphysis [8]. Our study demonstrated that there were strong correlations between the fracture height and the degree of bowing regardless of BPs medication. As the bowing deformity was larger, the site of the fracture in the femur was more distal.

The association between atypical femoral fractures and BPs therapy is controversial. BPs seem to increase risk of atypical fractures, with greater risk related to longer duration of management and declining risk after stopping BPs medication [11, 13]. However, atypical fractures have also seen observed in patients who have never been exposed to BPs [13, 28] and our findings add the evidence to these findings. Some authors have demonstrated that bisphosphonates did not delay spine fusion as compared to controls [29, 30]. Donnelly et al. [31] concluded that while BPs treatment may be an important risk factor for atypical fractures, it cannot be the sole risk factors. Thus, we believe that it is the nature of the fracture compared to the drug history that is more important.

This study has some limitations. First, this study was conducted with a retrospective nature, and therefore this design has inherent risk of observer bias, including the potential for missing data and inability to control confounding variables. Second, there were a limited number of patients. It is not easy to recruit a large sample size with a relatively rare injury. However, on investigation of the studies available regarding surgical outcome of such an injury, the current study is one of the largest ones. Third, we did not evaluate functional outcomes. Egol et al. [18] reported that 66% of patients with surgically treated complete atypical fractures became pain-free and pain combined with apprehension was a major cause of functional limitations. Lastly, the presence of osteomalacia may contribute to the findings of this study. More investigation on specific bone metabolism-related markers such as FGF23 level and associated genetic studies is not easily available under public health insurance system to which all of people should be affiliated.

In conclusion, of 76 patients (81 fractures), 54 (66.7%) fractures showed bony union within 6 months after the index surgery and 23 (28.4%) showed delayed union at a mean of 11.2 months (range, 8–18 months). There was no difference in healing rate between the BP group and the non-BP group. There were strong correlations between the fracture height and the degree of bowing both in the BP group and in the non-BP group. All fractures except 1 occurred at the diaphyseal region of the femur when not associated with BP medication.

## Figures and Tables

**Table 1:** Baseline patient characteristics.

	BP group (*n* = 73)	Non-BP group (*n* = 8)	*p*
Age (yr)	72.6 (58–85)	73.4 (61–84)	0.698
Female sex	73 (100%)	8 (100%)	1.000
Affected side (right : left)	35 : 38	4 : 4	1.000
Body mass index (kg/m^2^)	25.6 (17.3–40.7)	24.9 (21.2–32.3)	0.466
Bone mineral density (*T*-score, femur)	−2.2 (−0.6–−3.5)	−2.3 (−0.8–−3.7)	0.589
Comorbidities			
Cardiovascular disease	12 (16.4%)	2 (25.0%)	0.621
Cerebrovascular disease	5 (6.8%)	1 (12.5%)	0.475
Chronic pulmonary disease	8 (11.0%)	1 (12.5%)	1.000
Chronic renal disease	1 (1.4%)	0 (0.0%)	1.000
Chronic liver disease	5 (6.8%)	0 (0.0%)	1.000
Diabetes mellitus	17 (23.3%)	2 (25.0%)	1.000
Smoking	1 (1.4%)	0 (0.0%)	1.000
Parkinsonism	1 (1.4%)	1 (12.5%)	0.189
Cognitive impairment	3 (4.1%)	1 (12.5%)	0.346
ASA classification			0.216
I or II	66 (90.4%)	6 (75.0%)	
III or IV	7 (9.6%)	2 (25.0%)	
Koval score			1.000
1	23 (31.5%)	3 (37.5%)	
2 or 3	39 (53.4%)	4 (50.0%)	
≥4	11 (15.1%)	1 (12.5%)	
Presence of prodromal symptoms^†^			0.673
Yes	20 (27.4%)	1 (12.5%)	
No	53 (72.6%)	7 (87.5%)	

^†^Prodromal symptoms before fracture, such as thigh pain.

**Table 2:** Characteristics of fracture and surgery.

	BP group (*n* = 73)	Non-BP group (*n* = 8)	*p*
Injury mechanism			0.722
Fall-down	33 (45.2%)	3 (37.5%)	
Slip-down	35 (47.9%)	4 (50.0%)	
No trauma	5 (6.9%)	1 (12.5%)	
Fracture location			0.252
Subtrochanteric	27 (36.9%)	1 (12.5%)	
Diaphyseal	46 (63.1%)	7 (87.5%)	
Fracture height (%)	41.0 (17.2–66.3)	46.5 (20.1–54.2)	0.580
Fracture bilaterality			1.000
Complete or incomplete fracture in the contralateral side	18 (24.7%)	2 (25.0%)	
No fracture in the contralateral side	55 (75.3%)	6 (75.0%)	
Operation time (min)	118.7 ± 30.4 (55–280)	113.9 ± 34.2 (60–245)	0.823
The time interval from injury to operation (day)	4.2 ± 1.8 (1–8)	4.3 ± 1.6 (1–10)	0.702

**Table 3:** Characteristics of radiographic results.

	BP group (*n* = 73)	Non-BP group (*n* = 8)	*p*
Preoperative			
Femoral neck-shaft angle (°)	126.1 (119–137.3)	126 (121.6–136)	0.981
Coronal bowing of the femur (°)	10.3 (2.6–22.6)	10.6 (0.6–21.2)	0.994
Sagittal bowing of the femur (°)	13.3 (4.9–31.8)	13.4 (6–20.5)	0.758
Intraoperative			
Reduction in the coronal plane			0.828
Valgus	9 (12.3%)	1 (12.5%)	
Neutral	57 (78.1%)	6 (75.0%)	
Varus	7 (9.6%)	1 (12.5%)	
Reduction in the sagittal plane			0.628
Flexion	2 (2.8%)	0 (0.0%)	
Neutral	65 (89.0%)	7 (87.5%)	
Extension	6 (8.2%)	1 (12.5%)	
Postoperative			
Union within 6 months	49 (67.1%)	5 (63.5%)	1.000
Delayed union	20 (27.4%)	3 (37.5%)	0.682
Nonunion	4 (5.5%)	0 (0.0%)	1.000
Reoperation	3 (4.1%)	1 (12.5%)	0.346
Revision	3 (4.1%)	1 (12.5%)	0.346
